# Epigallocatechin-3-*O*-(3-*O*-methyl)-gallate-induced Differentiation of Human Keratinocytes Involves Klotho-Mediated Regulation of Protein Kinase-cAMP Responsive Element-Binding Protein Signaling

**DOI:** 10.3390/ijms15045749

**Published:** 2014-04-04

**Authors:** Hyoung-June Kim, Huikyoung Chang, Seung Hun Han, Min Seuk Lee, Ji-Yong Jung, SoonAe An, Seok-Yun Baek, Jin Ho Lee, John Hwan Lee, Tae Ryong Lee, Dong Wook Shin, Hongtae Kim

**Affiliations:** 1 Amorepacific Corporation R&D Center, 314-1, Bora-dong, Giheung-gu, Yongin-si, Gyeonggi-do 446-729, Korea; E-Mails: leojune@amorepacific.com (H.-J.K.); jhk282@gmail.com (H.C.); ninefog@amorepacific.com (J.-Y.J.); ananas@amorepacific.com (S.A.); sybaek@amorepacific.com (S.-Y.B.); johnlee@amorepacific.com (J.H.L.); trlee@amorepacific.com (T.R.L.); 2 Department of Biological Sciences, Sungkyunkwan University, 300, Cheoncheon-dong, Jangan-gu, Suwon 440-746, Korea; E-Mail: hun2130@naver.com; 3 Sulloccha Research Center, Jangwon Co., Ltd., Jeju 699-924, Korea; E-Mails: leems@jwgreent.co.kr (M.S.L.); jhlee@jwgreent.co.kr (J.H.L.); 4 School of Pharmacy, Sungkyunkwan University, Suwon 440-746, Korea

**Keywords:** (−)-epigallocatechin-3-*O*-gallate, methylated EGCG, klotho, keratinocyte differentiation

## Abstract

(−)-Epigallocatechin-3-*O*-gallate (EGCG) has long been known as a potent inducer of keratinocyte differentiation. Although its molecular mechanisms have been extensively studied, its actions on human skin remain to be elucidated. In this study, we demonstrated that methylated EGCG and EGCG increase the expression of klotho, and that klotho functions as a downstream target of EGCG and methylated EGCG in keratinocyte differentiation. We demonstrated that methylated EGCG3 and EGCG induce morphological changes in normal human epidermal keratinocytes (NHEKs) that are related to up-regulation of klotho expression. We also demonstrated that a klotho-induced keratinocyte differentiation marker in NHEKs is inhibited by H-89, a protein kinase (PKA) inhibitor. These results suggest that methylated EGCG and EGCG may function as inducers of keratinocyte differentiation via transcriptional regulation of the klotho protein.

## 1. Introduction

Keratinization of the epidermis is a well-defined program of differentiation: keratinocytes progress vertically from basal cells into spinous and granular cells to flattened, differentiated squamous cells in the stratum corneum [1]. The most important purpose of keratinocyte differentiation is the establishment and maintenance of the stratum corneum, the outermost layer of the skin. The stratum corneum consists of corneocytes and extracellular lipids secreted by differentiated keratinocytes, which together function as a barrier against the entry of chemicals and microbes from the environment and protect the body from dehydration [2].

The *klotho* (*KL*) gene, named after the Greek goddess said to spin the thread of life, was identified in 1997 as a gene mutated in the klotho mouse, which has an extremely short life span and suffers from multiple disorders resembling human premature-aging syndromes. It has been reported that the skin of *KL*^−^*^/^* ^−^ knockout mice has reduced dermal and epidermal thickness and a reduced number of hair follicles. However, little is known about KL regulation during the aging process in skin [3,4].

Tea (*Camellia sinensis* L.) is one of the most commonly consumed beverages in the world, particularly in Asian countries [5]. Green tea is known to have various bioactivities, including anti-carcinogenic [6,7], anti-mutagenic [8], anti-angiogenic [9], and anti-hypercholesterolemic [10] activities. (−)-Epigallocatechin-3-*O*-gallate (EGCG), the major catechin present in green tea, has been reported to induce differentiation and decreased cell proliferation in epidermal keratinocytes [11,12]. Based on extensively documented beneficial effects of EGCG on skin cells, many pharmaceutical and cosmetic companies are supplementing their skin care products with green tea extracts or EGCG [12–14].

**Figure 1.**
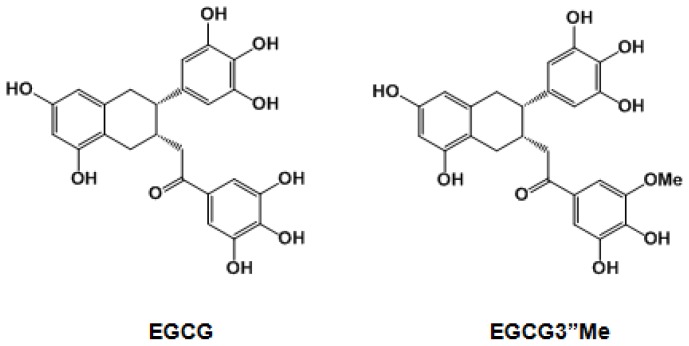
Chemical structures of epigallocatechin-3-*O*-gallate (EGCG) and epigallocatechin-3-*O*-(3-*O*-methyl)-gallate (EGCG″3Me).

Tea catechins undergo substantial biotransformation such as methylation, sulfation, and glucuronidation [15,16]. Figure 1 shows the structure of methylated EGCG in tea. Recent research has shown that *O*-methylated derivatives of EGCG inhibit type I atopic disorders (e.g., atopic dermatitis, allergic asthma and rhinitis) and type IV allergies (e.g., contact dermatitis, hypersensitivity pneumonitis and allograft rejection) more effectively than EGCG does [17,18]. However, the biological roles of methylated EGCG have been much less studied than those of EGCG.

In this study, based on the results of previous reports about EGCG-induced differentiation and reduced cell proliferation in epidermal keratinocytes, we have investigated and compared the biological effects of an *O*-methylated EGCG derivative and EGCG.

Here, we demonstrate that epigallocatechin-3-*O*-(3-*O*-methyl)-gallate (EGCG″3Me) activates the expression of *KL* gene products and induces the differentiation of human epidermal keratinocytes. We also found that the mechanism of EGCG″3Me-induced changes to skin barrier components was related to amplification of the KL signaling pathway, especially protein kinase A (PKA)-cAMP responsive element-binding protein (CREB) signal cascades.

## 2. Results

### 2.1. EGCG″3Me Induces Keratinocyte Differentiation

**Figure 2.**
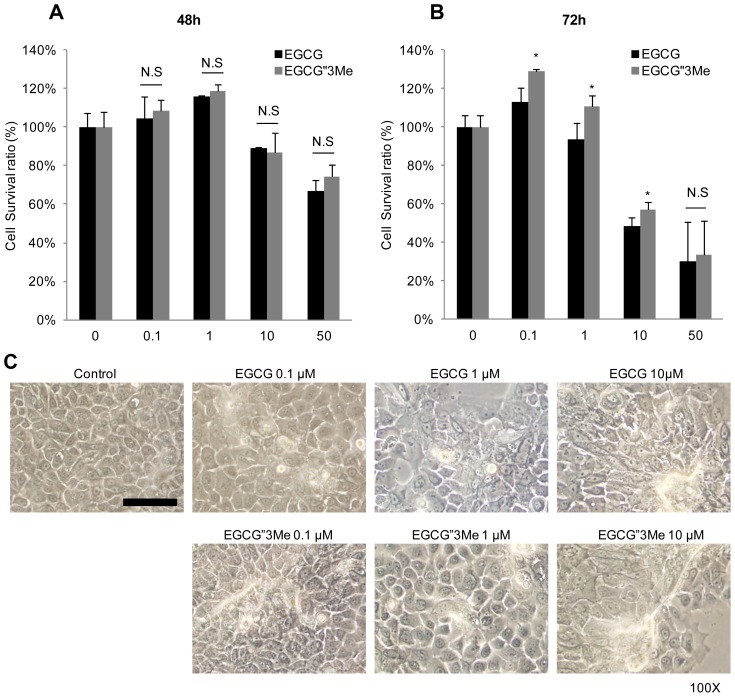
Epigallocatechin-3-*O*-(3-*O*-methyl)-gallate (EGCG″3Me) induces keratinocyte differentiation. Cell viability (measured using the Thiazolyl Blue Tetrazolium Blue assay) of normal human epidermal keratinocytes (NHEKs) after treatment with epigallocatechin-3-*O*-gallate (EGCG) and EGCG″3Me (0.1, 1, 10 and 50 μM) for (**A**) 48 h and (**B**) 72 h; (**C**) Morphological changes in NHEKs after treatment with various concentrations of EGCG and EGCG″3Me for 48 h, observed by phase contrast imaging (100× magnification, scale bar = 100 μm). N.S stands for not statistically significant. * *p* < 0.05 versus same dose of EGCG.

To investigate the effects of EGCG and EGCG″3Me on keratinocytes, we tested the cell viability of NHEKs after 48 and 72 h treatments with various concentrations of EGCG and EGCG″3Me. To get clear results of the differentiation makers, Keratin 10 (KRT10), Keratin 1 (KRT1) and involucrin (IVL) in Western blot analysis, we harvested the cells more than 2 days after and EGCG (Figure 2A). After 72 h, no toxicity was observed below 1 μM for each treatment, confluence (>90%) in NHEKs. To test cell survival ratio (%), we treated EGCG and EGCG″3Me from 0.1 to 50 μM in dose-dependent manner. After 48 h, no significant toxicity was observed for concentrations below 10 μM. Moreover, there were no significant differences between EGCG″3Me however, there were statistically significant differences in cell viability between EGCG and EGCG″3Me at 0.1, 1, and 10 μM, indicating that EGCG″3Me was less harmful to NHEKs than EGCG (Figure 2B). To study the effects of EGCG″3Me on keratinocyte differentiation, NHEKs were treated with EGCG″3Me, or with EGCG as a positive control. EGCG″3Me induced keratinocyte differentiation in a dose-dependent manner after 48 h (Figure 2C). After 48 h of treatment, both EGCG and EGCG″3Me induced morphological changes resulting in enlarged, flattened and squamous-like cells, which is a feature of differentiated keratinocytes (Figure 2C). These results indicated that the EGCG derivative EGCG″3Me was also capable of inducing keratinocyte differentiation.

### 2.2. EGCG″3Me Increases KL Expression

**Figure 3.**
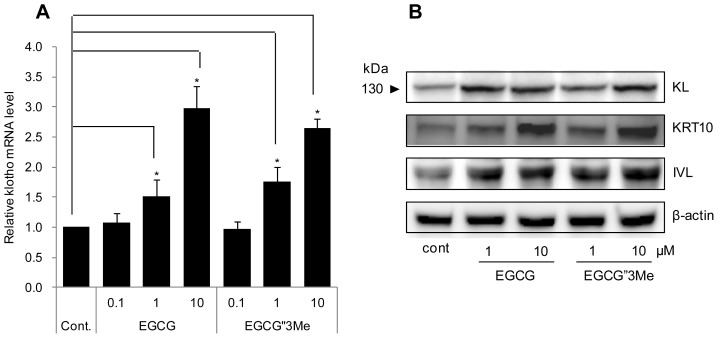
Epigallocatechin-3-*O*-gallate (EGCG) and epigallocatechin-3-*O*-(3-*O*-methyl)-gallate (EGCG″3Me) increase klotho (KL) expression. (**A**) Normal human epidermal keratinocytes (NHEKs) analyzed by qRT-PCR 24 h after treatment with EGCG and EGCG″3Me (0.1, 1, and 10 μM). The values represent the mean ± standard deviation of three independent experiments (* *p* < 0.05); and (**B**) The expression level of KL, keratin 10 (KRT10), involucrin (IVL) and β-actin in NHEKs analyzed by western blot 24 h after treatment with EGCG and EGCG″3Me (1 and 10 μM). The results are representative of three independent experiments.

To study the effects of EGCG and EGCG″3Me on *KL* expression at the transcription level, we used qRT-PCR to analyze NHEKs treated with EGCG or EGCG″3Me. The analysis showed a dose-dependent increase in the expression of KL mRNA in cells treated with EGCG or EGCG″3Me (Figure 3A). EGCG and EGCG″3Me (1 and 10 μM) increased the expression of *KL* mRNA in NHEKs by 3- and 2.5-fold, respectively. A corresponding increase in KL protein expression was confirmed by Western blot analysis of cell lysates from control cells or NHEKs treated with EGCG or EGCG″3Me (Figure 3B). β-actin was used as a loading control. Moreover, keratinocyte differentiation markers such as keratin 10 (KRT10) and IVL were also up-regulated by EGCG and EGCG″3Me.

### 2.3. KL Increases Keratinocyte Differentiation Markers

**Figure 4.**
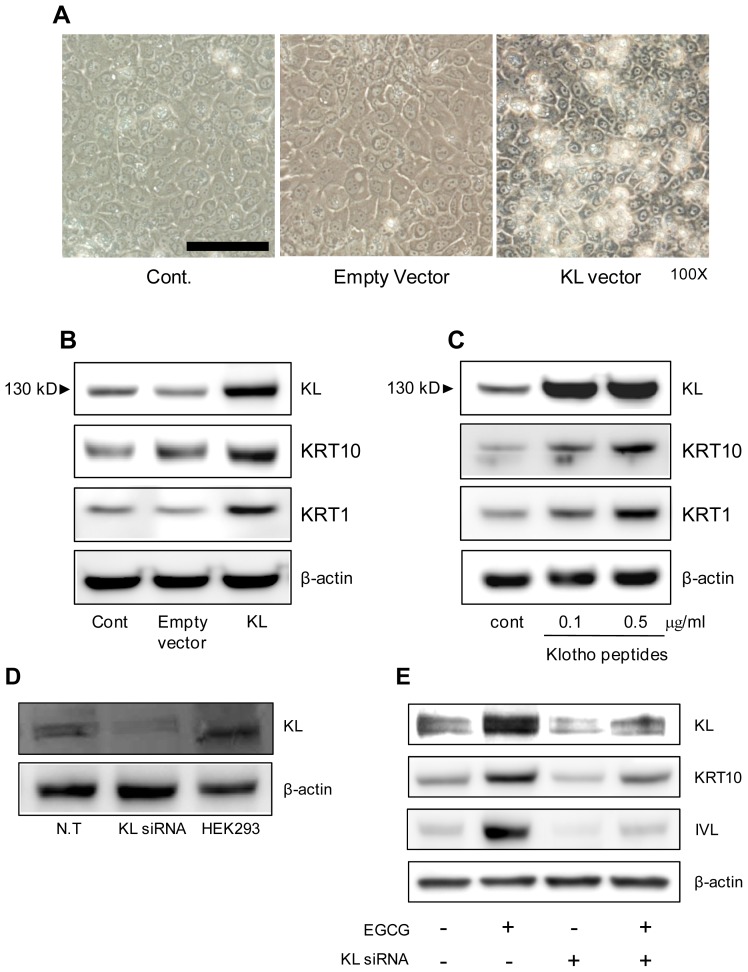
Klotho (KL) increases keratinocyte differentiation markers. (**A**) Morphology of normal human epidermal keratinocytes (NHEKs) transfected for 48 h with plasmids encoding KL, observed by phase contrast imaging (100× magnification; scale bar = 100 μm); (**B**) Protein levels in NHEKs after ectopic over-expression of Myc-KL, KL, keratin 10 (KRT10), keratin 1 (KRT1) and β-actin, analyzed by western blot analysis; (**C**) NHEKs treated with KL peptides for 48 h and analyzed by western blot using antibodies against KL, KRT1, KRT10, and β-actin. The results are representative of three independent experiments.; (**D**) KL expression in NHEKs transfected with KL siRNA or non-targeting siRNA for 48 h, compared with KL expression in HEK293 cells, using western blot analysis; and (**E**) NHEKs were transfected for 48 h with KL siRNA and analyzed for KL, KRT10, involucrin (IVL) and β-actin by western blot. The results are representative of three independent experiments.

Since EGCG, an inducer of keratinocyte differentiation, increases KL expression and keratinocyte differentiation markers, we investigated whether KL expression can regulate keratinocyte differentiation. As shown in Figure 4A, NHEKs transfected with plasmids encoding KL (mammalian expression vectors) showed the morphology of keratinocytes, including enlarged, flattened, and squamous-like cells with lamellar granules, in contrast to cells transfected with empty vector. Consistent with this result, Figure 4B,C show that ectopic expression of KL or addition of KL peptides, respectively, increased the keratinocyte differentiation markers KRT10 and KRT1. We also compared KL expression levels in normal NHEKs and HEK293 cells and NHEKs knocked-down for KL (Figure 4D). We observed that the up-regulation of KRT10 and IVL by the EGCG treatment was attenuated in cells transfected with KL siRNA (Figure 4E). These results indicate that KL is deeply involved in keratinocyte differentiation.

### 2.4. Morphological Changes in NHEKs Induced by EGCG and EGCG″3Me Involve PKA Signaling and Are Inhibited by the PKA Inhibitor H-89

**Figure 5.**
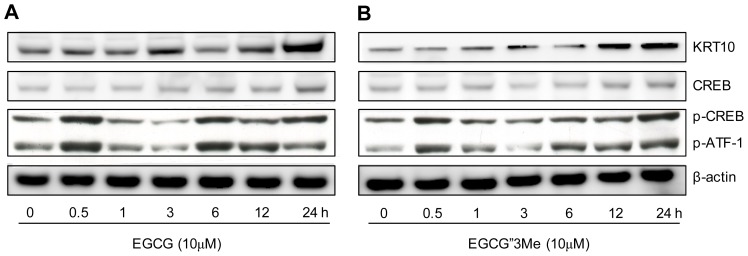

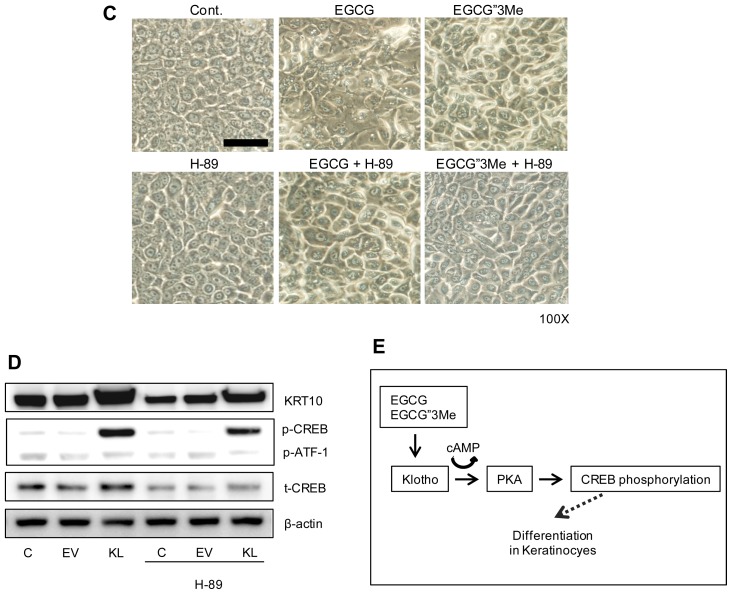
Epigallocatechin-3-*O*-gallate (EGCG) and epigallocatechin-3-*O*-(3-*O*-methyl)-gallate (EGCG″3Me) can induce phosphorylation of cAMP responsive element-binding protein (CREB). Normal human epidermal keratinocytes (NHEKs) were treated with (**A**) EGCG (10 μM) or (**B**) EGCG″3Me (10 μM) for 0, 0.5, 1, 3, 6, 12 or 24 h and analyzed by Western blotting to detect the expression levels of total CREB, phosphorylated CREB (pCREB), and phosphorylated activating transcription factor 1 (pATF1). β-actin was used as a loading control; (**C**) After pre-treatment with DMSO or the protein kinase (PKA) inhibitor H-89, NHEK cells were treated with EGCG or EGCG″3Me for 48 h and analyzed for keratinocyte differentiation (scale bar = 100 μm); (**D**) NHEKs were transfected with over-expression vectors of KL or H-89 for 24 h and analyzed for KRT10, phosphorylated CREB, total CREB, phosphorylated activating transcription factor 1 (pATF1) and β-actin by western blot (c: control; EV: empty vector; KL: vector containing *KL* gene). The results are representative of three independent experiments; and (**E**) Graphic view of signaling pathway.

We tested whether EGCG″3Me could induce changes in NHEKs via the PKA pathway. After treatment of NHEKs with EGCG or EGCG″3Me (Figure 5A,B, respectively) for 12 h, KRT10 expression levels started to increase. We then investigated whether the phosphorylation level of CREB, which is activated by PKA via the cAMP signaling pathway, was altered after the treatment with EGCG or EGCG″3Me. After 6 h, the level of phosphorylated CREB was strongly increased, however, the total level of CREB was largely unchanged or slightly increased after 24 h. We also tested whether EGCG″3Me could induce morphological changes in NHEKs via the PKA pathway. As shown in Figure 5C, EGCG″3Me-induced morphological changes to NHEKs were dramatically inhibited by pre-treatment with H-89, a highly selective inhibitor of cAMP-dependent PKA. H-89 also dramatically inhibited the expression of KL-induced KRT10, a representative differentiation marker in keratinocytes, and reduced the level of CREB phosphorylation in NHEK cells (Figure 5D).

These results indicate that EGCG and EGCG″3Me are deeply involved in KL-related cellular actions through the PKA-CREB signaling pathway, which can induce keratinocyte differentiation.

## 3. Discussion

In this study, we show results indicating a correlation between EGCG″3Me and KL in keratinocyte differentiation. We found that KL was up-regulated by EGCG″3Me at the transcriptional and translational level, influencing keratinocyte differentiation though the PKA-CREB signaling pathway. Although research has shown that EGCG exerts photo-protective and anti-carcinogenic effects in skin [19], its molecular mechanisms relating to keratinocyte differentiation remain to be fully elucidated, especially the mechanisms of EGCG derivatives such as methylated EGCG.

Until recently, little has been known about the cellular activities of EGCG derivatives, including EGCG″3Me. To reveal their safety and efficacy, the cellular effect and biological roles of these derivatives should be studied and compared with the established effects of EGCG. Therefore, EGCG was used as a positive control in all of the experiments in this study. As shown in Figure 2, we compared the cell viability of NHEKs treated with EGCG″3Me and EGCG for 48 h at concentrations up to 10 μM. At these conditions, EGCG″3Me induced keratinocyte differentiation at the same level as EGCG, which is known to induce keratinocyte differentiation [20]. We also found that EGCG″3Me could increase the KL expression level in NHEKs. In another study, KL was found to be related to resistance against H_2_O_2_-induced oxidative stress damage in HaCaT cells (immortalized human keratinocytes) and NHEKs [21]. Here, we show that when the level of KL was transiently reduced, the levels of EGCG″3Me-mediated KRT10 and IVL were also reduced. In contrast, when KL was over-expressed, differentiation markers were up-regulated (Figure 3E). These results indicate that the differentiating activities of EGCG″3Me are deeply related to increased KL expression and may have a role in KL-related signaling cascades. However, the mechanism by which EGCG″3Me up-regulates KL expression remains to be elucidated.

Interestingly, the treatment of NHEKs with KL peptide also increased the level of differentiation markers and morphological changes, similar to the over-expression of KL by transient transfection. KL is known as a protein that functions as an anti-aging hormone [22]. Because of its hormonal property, it might regulate itself at the cellular level via a positive feedback mechanism. To investigate the signaling pathway by which EGCG″3Me or KL may increase keratinocyte differentiation, we speculated and observed that EGCG″3Me and KL were related to the PKA-CREB pathway in NHEKs. After PKA is activated by cAMP [23], PKA directly activates CREB [24,25]. In the HaCaT cell line, the secondary messenger cAMP is important for inducing terminal differentiation markers [26]. Moreover, KL expression is mediated through PKA-dependent phosphorylation of the transcription factor CREB in retinal pigment epithelium (RPE) and endothelial cells [27,28]. It has previously been reported that an increase in extracellular or intracellular calcium levels cause keratinocyte differentiation [29–32]. However, because EGCG could not induce the uptake or mobilization of intracellular calcium levels in NHEKs [33], we focused on the signal cascades from EGCG″3Me to the KL and PKA-CREB pathway. We found that the phosphorylation of CREB increased upon treatment of cells with EGCG″3Me or EGCG (Figure 4A,B), and that pre-treatment with the PKA inhibitor H-89 inhibited the EGCG″3Me (or EGCG) and over-expression of KL-induced keratinocyte differentiation markers (Figure 5C,D). In addition, the reduction of KL-mediated phosphorylation of CREB was seen after H-89 pre-treatment for both the EGCG″3Me (or EGCG) and KL over-expression treatment. Finally, it might be postulated that the increase in cAMP and KL expression by EGCG″3Me is the result of PKA activation and induce the phosphorylation of CREB (Figure 5). These data lead us to conclude that KL is a potent regulator that activates PKA signaling in EGCG″3Me (or EGCG) mediated keratinocyte differentiation.

## 4. Experimental Procedures

### 4.1. Materials

EGCG (purity > 97%) was purchased from Morechem (Seoul, Korea). EGCG″3Me was obtained from Nagara Science Co., Ltd., (Gifu, Japan). EGCG and EGCG″3Me were dissolved in dimethyl sulfoxide (DMSO) and filter-sterilized before use. Normal human epidermal keratinocytes (NHEKs) were purchased from Lonza (Allendale, NJ, USA). Human embryonic kidney (HEK) 293T cells were purchased from the American Type Culture Collection (Manassas, VA, USA). KL peptide was purchased from R&D systems (Minneapolis, MN, USA). H-89 was obtained from Calbiochem (San Diego, CA, USA) and dissolved in DMSO.

### 4.2. Cell Culture

HEK 293T cells were maintained in Dulbecco’s Modified Eagle Medium (DMEM) supplemented with 10% fetal bovine serum (FBS) at 37 °C in 5% CO_2_ (*v*/*v*). NHEKs were cultured in Keratinocyte Growth Medium (KGM-GOLD, Lonza, Allendale, NJ, USA). NHEKs were subcultured by detachment with 0.025% trypsin and transfer into new tissue culture flasks. Cells were used within passage 2.

### 4.3. Cell Viability Assay

After treatment of NHEKs with EGCG″3Me and EGCG (0, 0.1, 1, 10 and 50 μM) for 48 and 72 h, 50 μL (2 mg/mL) of Thiazolyl Blue Tetrazolium Bromide (MTT, Sigma-Aldrich, St. Louis, MO, USA) dissolved in KGM-GOLD was added to the cells, which were then incubated for 3 h at 37 °C. The medium was removed, and formazan crystals of the cells were solubilized in 200 μL of DMSO by gentle shaking for 10 min. The amount of formazan present was quantified using a microplate reader (Molecular Devices, Sunnyvale, CA, USA) at 540 nm.

### 4.4. RNA Extraction and Quantitative Real-Time RT-PCR (qRT-PCR)

Total RNA was isolated using TRIzol™ (Invitrogen, Carlsbad, CA, USA), according to the manufacturer’s protocol. The RNA concentration was measured spectrophotometrically, and RNA integrity was assessed using a BioAnalyzer 2100 (Agilent Technologies, Santa Clara, CA, USA). Four micrograms of RNA was reverse-transcribed into cDNA with SuperScript^®^III reverse transcriptase (Invitrogen, Carlsbad, CA, USA), and aliquots were stored at −70 °C. The expression levels of target genes were determined using quantitative real-time TaqMan RT-PCR technology (7500Fast, Applied Biosystems, Foster City, CA, USA). The following cycling conditions were used: 95 °C for 10 min, 50 cycles of 95 °C for 15 s, and 60 °C for 1 min. The following TaqMan probes were used: KL, Hs00183100_m1; and RPL13A, Hs04194366_g1 (Applied Biosystems, Foster City, CA, USA), which is commonly used a housekeeping gene for normalization in NHEKs and was used to normalize the variations in cDNA levels.

### 4.5. Knock-Down or Over-Expression of KL

Briefly, to knock down KL, NHEKs were transfected for 48 h with 50 nM of ON-targetplus SMARTPOOL siRNA (Non-targeting#2, #D-001810-02; and human KL, #L-011936-01-0020) according to the manufacturer’s protocol (Thermo Fisher Scientific, Lafayette, CO, USA) using Lipofectamine^®^ RNAi MAX Transfection Reagent (Invitrogen, Carlsbad, CA, USA). For *KL* over-expression, using mammalian expression vectors encoding the *KL* gene, 1 μg/mL of plasmid was transfected for 48 h using the X-tremeGENE HP DNA Transfection Reagent (Roche Diagnostics GmbH, Mannheim, Germany).

### 4.6. Western Blot Analysis

NHEKs were lysed with RIPA cell lysis buffer containing a protease inhibitor and phosphatase inhibitor cocktails (Sigma, St. Louis, MO, USA). After centrifugation of the lysate at 15,000× *g* for 20 min, the supernatant was used to analyze the level of proteins. The concentration of proteins was determined using the Bradford method with bovine serum albumin (BSA) as a standard. Protein samples (40 μg) were separated by sodium dodecyl sulfate polyacrylamide gel electrophoresis (SDS-PAGE), and the gel was then transferred onto a polyvinylidene difluoride (PVDF) membrane. Membranes were blocked with 5% non-fat skim milk in TBS-T buffer (10 mM Tris-HCl at pH 8.0, 150 mM NaCl, 0.015% Tween-20) for 1 h at room temperature. Blocked membranes were probed overnight at 4 °C with anti-KL antibody (Cosmo Bio, Tokyo, Japan); anti-keratin 10 (KRT10) and anti-keratin 1 (KRT1) (Covance, Princeton, NJ, USA); involucrin (IVL), CREB, phosphorylated CREB and β-actin (Cell Signaling, Boston, MA, USA). Blots were washed at least three times in TBS-T buffer and reacted with horseradish peroxidase-conjugated anti-mouse or anti-rabbit IgG secondary antibodies raised in goat (Bio-Rad, Hercules, CA, USA) at room temperature for 1 h. Blots were developed with ECL solution (Amersham Pharmacia Biotech, Piscataway, NJ, USA).

### 4.7. Statistical Analyses

Statistical analyses were performed using Student’s *t*-test. All measurements were obtained from at least three independent experiments carried out in triplicate, and values are expressed as the mean ± standard deviation.

## 5. Conclusions

In conclusion, the identification of KL as a new target molecule for EGCG″3Me (or EGCG) provides new insight into the interplay between signaling pathways and protein networks in keratinocyte differentiation.
